# Supplementary material for: Review of Antimicrobial Resistance in Wastewater in Japan: Current Challenges and Future Perspectives
Source: Antibiotics (Basel). 2022 Jun 24;11(7):849. doi: 10.3390/antibiotics11070849 (PMC9312076; doi:10.3390/antibiotics11070849)
Supplement: Supplementary file 1 [file antibiotics-11-00849-s001.zip › antibiotics-1757281-supplementary.pdf]

Supplementary Material S1. Search terms of literature released on PubMed (A) and Igaku Chuo Zashi (ICHUSHI) database (B).

(A) Search terms on PubMed:

‘japan’[affiliation] AND ‘antimicrobial’[All Fields] AND ‘resistance’[All Fields] AND (‘wastewater’[All Fields] OR ‘sewage’[All Fields])

(B) Search terms on ICHUSHI:

(((下水/TH or 下水/AL) or (汚水/TH or 排水/AL)) and (抗感染剤/TH or 抗菌薬/AL) and 耐性/AL) and (PT=原著論文)

‘下水’ means sewage; ‘汚水’ and ‘排水’ mean wastewater; ‘抗感染剤’ and ‘抗菌薬’ mean antimicrobials; ‘耐性’ mean resistance; ‘原著論文’ mean original article. TH, thesaurus; AL, all fields; PT, publication type
